# Transcriptome analysis revealed potential genes involved in thermogenesis in muscle tissue in cold-exposed lambs

**DOI:** 10.3389/fgene.2022.1017458

**Published:** 2022-10-21

**Authors:** Kaixi Ji, Dan Jiao, Guo Yang, Abraham Allan Degen, Jianwei Zhou, Hu Liu, Wenqiang Wang, Haitao Cong

**Affiliations:** ^1^ Key Laboratory of Stress Physiology and Ecology of Gansu Province, Northwest Institute of Eco-Environment and Resources, Chinese Academy of Sciences, Lanzhou, China; ^2^ University of Chinese Academy of Sciences, Beijing, China; ^3^ Desert Animal Adaptations and Husbandry, Wyler Department of Dryland Agriculture, Blaustein Institutes for Desert Research, Ben-Gurion University of Negev, Beer Sheva, Israel; ^4^ State Key Laboratory of Grassland and Agro-Ecosystems, College of Pastoral Agriculture Science and Technology, Lanzhou University, Lanzhou, China; ^5^ College of Ecology, Lanzhou University, Lanzhou, China; ^6^ Dongying Modern Animal Husbandry Development Service Center, Dongying, China

**Keywords:** cold exposure, thermogenesis, muscle tissue, sheep, transcriptome sequencing (RNA-seq)

## Abstract

Cold tolerance is an important trait for sheep raised at high altitudes. Muscle tissue, comprising 30–40% of the total body mass, produces heat during cold exposure. However, little is known about the genetic mechanisms of this tissue and its role in thermogenesis in lambs. We examined genes in skeletal muscle tissue in a cold-adapted sheep breed, Altay, and a cold-intolerant sheep breed, Hu, when exposed to low air temperature. Three ewe-lambs of each breed were maintained at −5°C and three ewe-lambs of each breed were maintained at 20°C. After cold exposure for 25 days, the *longissimus dorsi* of each lamb was collected, and transcriptome profiles were sequenced and analyzed. The results of RNA-seq showed that the average reads among the four groups were 11.0 Gbase. The genome mapping rate averaged 88.1% and the gene mapping rate averaged 82.5%. The analysis of differentially expressed genes (DEGs) indicated that the peroxisome proliferator-activated receptors (PPAR), cAMP, and calcium signaling pathways and muscle contraction in muscle tissue were linked to thermogenesis in cold-exposed lambs. Furthermore, *PCK1* (*phosphoenolpyruvate carboxykinase1*) increased glyceroneogenesis in cold-exposed Altay lambs, and *APOC3* (*apolipoprotein C3*), *LPL* (*lipoprotein lipase*), and *FABP4* (*fatty acid binding protein 4, adipocyte*) were involved in the intake and transport of free fatty acids. In Hu sheep, cAMP biosynthesis from ATP hydrolysis was regulated by *ADCY10* (*adenylate cyclase*) and *ADORA2a* (*adenosine A2a receptor*). Skeletal muscle contraction was regulated by *MYL2* (*myosin light chain 2*). In conclusion, cold exposure altered the expression level of genes involved in heat production in muscle tissue. Some potential mechanisms were revealed, including calcium ion transport in the calcium signaling pathway, fatty acid metabolism in the PPAR signaling pathway, and cAMP biosynthesis in the cAMP signaling pathway. This study implied that skeletal muscle plays an important role in thermoregulation in lambs.

## 1 Introduction

Cold stress has negative effects on livestock production. For example, feed digestibility is reduced, respiratory disorders are increased, and blood-flow distribution is affected in ruminants (Degen and Young, 1981, 1980; Christopherson and Kennedy, 1983; Young, 1983; Chase, 2011). Neonatal lambs are particularly vulnerable, as severe cold stress can cause high mortality (Young, 1983; Stott and Slee, 1985), especially in the northern mountain region, where the ambient temperature reaches −35°C to −50°C in winter (Xia, 2011).

The capacity of heat production (HP) is critical for animals to maintain core body temperature and prevent hypothermia when exposed to cold. Both shivering thermogenesis (ST) and non-shivering thermogenesis (NST) increase HP in cold-exposed animals. ST generally occurs in the initial stage of cold exposure, and is related to actin, myosin, and hydrolysis of ATP (Blondin et al., 2014; Squire et al., 2017). With prolonged cold exposure, animals switch from ST to NST. Two NST mechanisms have been researched extensively (Fuller-Jackson and Henry, 2018; Roesler and Kazak, 2020; Grigg et al., 2022): 1) uncoupling process of substrate oxidative phosphorylation in mitochondria, namely UCP1 (uncoupling protein-1) dependent thermogenesis, which exists primarily in brown adipose tissue (BAT) for cold-induced humans and mice (Mueez et al., 2018; Okamatsu-Ogura et al., 2020); and 2) calcium ion transport between sarcoplasmic reticulum (SR) and cellular matrix in muscle, especially for BAT-lacking birds, marsupial and neonatal animals (Grigg et al., 2022). The Ca^2+^-leakage is mediated by sarcoplasmic reticulum Ca^2+^-ATPase (SERCA), ryanodine receptor 1 (RyR1), sarcolipin (SLN) and other factors (Bal et al., 2018; Meizoso-Huesca et al., 2022). In a study of lambs, ST and NST were triggered shortly after the onset of cold exposure and accounted for 46% and 31%, respectively, of summit metabolism (Alexander and Williams, 1968). A better understanding of thermogenesis in response to cold could be beneficial in improving sheep welfare and reducing economic losses. To date, however, the molecular mechanisms in sheep in response to cold are still unclear.

Obese sheep were reported to have lower heat production, energy expenditure, and the expression of *UCP1* in retroperitoneal adipose tissue than lean sheep (Henry et al., 2008; Henry et al., 2015). Altay is a fat-tailed sheep breed from Xinjiang Province, China, well-adapted to cold. The daily mean temperature in Xinjiang is -16.3°C (Yang, 2015). Hu is a lean sheep breed originating from southern China and has low resistance to cold. The average annual temperature in southern China is 16°C (Han, 2016). Genes involved in thermoregulation in liver (Jiao et al., 2021a) and adipose tissue (Jiao et al., 2021b) were reported for Altay and Hu sheep when exposed to cold. In the current study, we examined the genetic mechanisms in muscle tissue in thermoregulation in these sheep breeds by employing transcriptome profiling, qRT-PCR, gene ontology (GO) analyses and Kyoto Encyclopaedia of Genes and Genomes (KEGG) pathways analyses.

## 2 Materials and methods

### 2.1 Animals and diets

Six Altay (32.0 ± 2.6 kg) and six Hu (31.8 ± 2.7 kg) ewe-lambs, 6-month of age, were used in this study. All lambs were maintained individually in metabolic cages (1.2 m × 0.6 m × 1.8 m), and offered alfalfa pellets (12.4 MJ metabolizable energy and 148 g/kg crude protein on dry matter basis) *ad-libitum* (Zhou et al., 2020), with free access to water. The chemical composition of the alfalfa pellets is presented in Supplementary Table S1 of supplementary File1.

### 2.2 Air temperature adjustment profiles

All lambs were kept at 20°C for 7 days to adapt to the conditions, and then were divided into four groups. Three-Altay lambs (AM1) and three Hu lambs (HM1) were kept in a room in which the temperature was lowered from 20°C to −5°C by reducing the temperature by 5°C every 2 days for 25 days (Jiao et al., 2021b). The other three Altay lambs (AM2) and three Hu lambs (HM2) were kept in a temperature-controlled room at 20°C during the whole experimental period. Rectal temperature was measured using a mercury thermometer at 06:00, 14:00, and 22:00 before slaughter.

### 2.3 Sample collections

On day 36, the lambs were euthanized with an intravenous injection of 20% sodium pentobarbital (Sigma, St. Louis, MO, United States). Then the *longissimus dorsi* muscle tissue was collected from each lamb and placed in a 5 ml Eppendorf tube filled with RNAlater (Merck KGaA, Darmstadt, Germany) to prevent the degradation of RNA from degrading. All samples were snap-frozen immediately in liquid nitrogen and then stored at −80°C for analysis.

### 2.4 Total RNA extraction, library construction, and sequencing

Total RNA from each muscle was extracted using Trizol reagent (Invitrogen, Carlsbad, CA, United States) according to the manufacturer’s instructions. The concentration and integrity of total RNA were assessed using the Agilent 2100 Bioanalyzer (Agilent Technologies, Santa Clara, CA, United States). All RNA samples were screened at the RNA Integrity Number (RIN) > 7.0 and the 28S/18S rRNA ratio > 1.0.

The poly-A mRNA was isolated from the total RNA using OligodT magnetic beads (Takara, Kusatsu, Japan) and fragmented using RNA fragmentation kits (Tiangen Biotech, Beijing, China). The first-strand cDNA was synthesized from the fragmented mRNA using random oligonucleotide primers and reverse transcriptase (Tiangen Biotech, Beijing, China). The synthesis of the second cDNA usd DNA polymerase I and RNAase Ⅱ treatments. The cDNA fragments produced had a single ‘A' nucleotide base added, followed by ligation of an adapter. The products were then purified and enriched with PCR amplification to create the final cDNA library.

### 2.5 Gene expression analyses

The raw reads were filtered with quality control software of SOAPnuke (BGI, Shenzhen, China) to exclude low quality reads (more than 20% of bases in the total reads had a quality score lower than 15), adaptor reads (reads with joint contamination), and highly unknown base N content (total number of reads which contain more than 5% unknown N bases). Clean reads obtained were aligned to the reference genome of *Ovis aries* (Oar_v4.0; https://www.ncbi.nlm.nih.gov/assembly/GCF_000298735.2/) using the HISAT alignment tool (Centre for Computational Biology, Johns Hopkins University, MD, United States). The expression levels of genes were calculated and assessed based on the FPKM (fragments per kilobase of exon model per million reads mapped) value.

### 2.6 Function enrichment and protein-protein interaction network analyses

Gene ontology analyses was used to analyze the functions of differentially expressed genes (DEGs). Significantly changed GO terms were obtained by mapping DEGs to the online GO database (http://www.geneontology.org/) with a threshold of adjusted *p* ≤ 0.05.

The KEGG pathway were analyzed based on the online database (http://www.kegg.jp/kegg/pathway.html/). The significantly enriched pathways for DEGs were screened according to the adjusted *p* ≤ 0.05.

The PPI network and regulatory network were analyzed using Cytoscape 3.6.1 (Cytoscape Consortium, San Diego, CA, United States).

### 2.7 RNA-seq validation

To validate whether the RNA-seq results were reliable, the relative expression levels of four identified DEGs, *lipoprotein lipase* (*LPL*), *troponin C1*, *slow skeletal and cardiac type* (*TNNC1*), *fatty acid binding protein 4*, *adipocyte* (*FABP4*), and *parvalbumin* (*PVALB*), were verified using quantitative real-time PCR (qRT-PCR). The primers were designed using Primer Premier 6 (PREMIER Biosoft, San Francisco, CA, United States) (Table 1), and the *β-actin* gene was used as a housekeeping gene to standardize the levels of gene expression. The cDNA amplifications used PrimerScript RT reagent kits (Takara, Kyoto, Japan) with gDNA Erase (Takara, Kyoto, Japan), according to the manufacturer’s instructions. The qPCR reaction contained 2 µl of cDNA, 0.8 µl of each forward and reverse primers (10 um/ul), 10 µl of TB GreenTM Premix Ex Taq II (Takara, Kyoto, Japan), 6 µl of RNAase free water, and 0.4 µl of ROX Reference Dye II (50×). The qPCR reaction used an Aligent Mx3000P system (Agilent Technologies, Santa Clara, CA, United States), and a two-step amplification method: 1) a pre-degeneration of 15 s at 95°C; and 2) 5 s at 95°C and 34 s at Tm for 40 cycles. Cycle threshold (CT) values were recorded and relative expressions were determined according to the 2^-△△ct^ method (Livak and Schmittgen, 2001).

**TABLE 1:** Primer sequence used for qRT-PCR.

Gene symbol	Primer sequences (5′–3′)	Product length (bp)	Tm (°C)
*LPL*	CGA​CAG​GAT​TAC​AAG​AGG​AA	100	60
	AGGAATGAGGTGGCAAGT		
*TNNC1*	ATG​ATT​GAC​GAG​GTG​GAT​G	131	61
	ATGCGGAAGAGGTCTGAA		
*FABP4*	AGA​TGA​AGG​TGC​TCT​GGT​A	137	60
	ATG​CTC​TCT​CGT​AAA​CTC​TG		
*PVALB*	GCT​GAG​GAC​ATC​AAG​AAG​G	180	60
	GACAGGTCTCTGGCATCT		
*β-actin*	AGC​CTT​CCT​TCC​TGG​GCA​TGG​A	113	60
	GGA​CAG​CAC​CGT​GTT​GGC​GTA​GA		

### 2.8 Statistical methods

The DEGs were determined by pairwise comparisons (Li et al., 2018; Jiao et al., 2021b) using R version 4.2.1. The fold changes (FC) were calculated as: FC = $\frac{avg\;FPKM\;\left(cold\right)\;}{avg\;FPKM\;\left(thermoneutral\right)}$ ) or FC = $\frac{avg\;FPKM\;\left(Altay\right)}{avg\;FPKM\;\left(Hu\right)}$ . The significant differences in gene expressions were judged as a standard on the adjusted *p* (Q-value) ≤ 0.001 and |log2FC| ≥ 1. The adjusted *p* value is similar to the *p* value, except it is a measure of significance in terms of the false discovery rate rather than the false positive rate, and it effectively avoids some null hypotheses with significant features (Storey and Tibshirani, 2003). The adjusted *p* value was corrected by using an R package (Wang et al., 2010).

The difference in the relative expression of candidate genes was determined by a paired t-test (SPSS, v.24.0, IBM SPSS Statistics, IBM Corporation). Significance was accepted at a *p* ≤ 0.05. Results are presented as means ± SEM.

## 3 Results

All lambs appeared to be healthy throughout the study. The cold-exposed Altay and Hu lambs increased their rectal temperatures (Supplementary Table S2 in supplementary file 1).

### 3.1 Sequencing and mapping

Twelve cDNA libraries were attained and sequenced from the Altay and Hu lambs. A summary of the transcriptome sequencing is presented in supplementary file 2. The average total clean bases among these cDNA profiles were about 11.0 Gb (Table 2). After filtering the low-quality raw reads, the average ratio of clean reads for AM1, AM2, HM1, and HM2 lambs were 95.2%, 93.3%, 95.2%, and 94.4%, respectively. The average mapping ratio of clean reads to the genome were 88.2%, 87.9%, 88.3%, 88.0%, and to the gene were 83.1%, 82.7%, 82.3%, 82.0%, respectively (Table 2).

**TABLE 2:** Statistics of total reads and mapping ratio to reference genome.

Groups	Samples	Clean base	Raw reads	Clean reads	Mapping ratio to genome (%)	Mapping ratio to gene (%)
AM1	AM1_1	11118591400	117046090	111185914	89.38	84.07
	AM1_2	11115941200	117046038	111159412	87.72	82.19
	AM1_3	10955440800	114551126	109554408	87.47	82.91
AM2	AM2_1	10991787200	117040298	109917872	88.22	82.96
	AM2_2	10930327200	119383422	109303272	87.33	82.19
	AM2_3	11040183600	117040472	110401836	88.21	82.90
HM1	HM1_1	11062332400	117046340	110623324	87.18	81.66
	HM1_2	10944857600	114555576	109448576	88.59	82.28
	HM1_3	10948947400	114555876	109489474	89.17	82.90
HM2	HM2_1	10974164200	117039644	109741642	87.11	81.62
	HM2_2	10974702200	116959254	109747022	88.65	81.91
	HM2_3	10948267800	114556014	109482678	88.09	82.40

AM1: Altay lambs at −5°C, AM2: Altay lambs at 20°C and HM1: Hu lambs at −5°C; HM2: Hu lambs at 20°C.

AM1_1, AM1_2, AM1_3, AM2_1, AM2_2, AM2_3, HM1_1, HM1_2, HM1_3, HM2_1, HM2_2, and HM2_3 represent twelve individuals.

### 3.2 Gene expression and annotation

**FIGURE 1:**
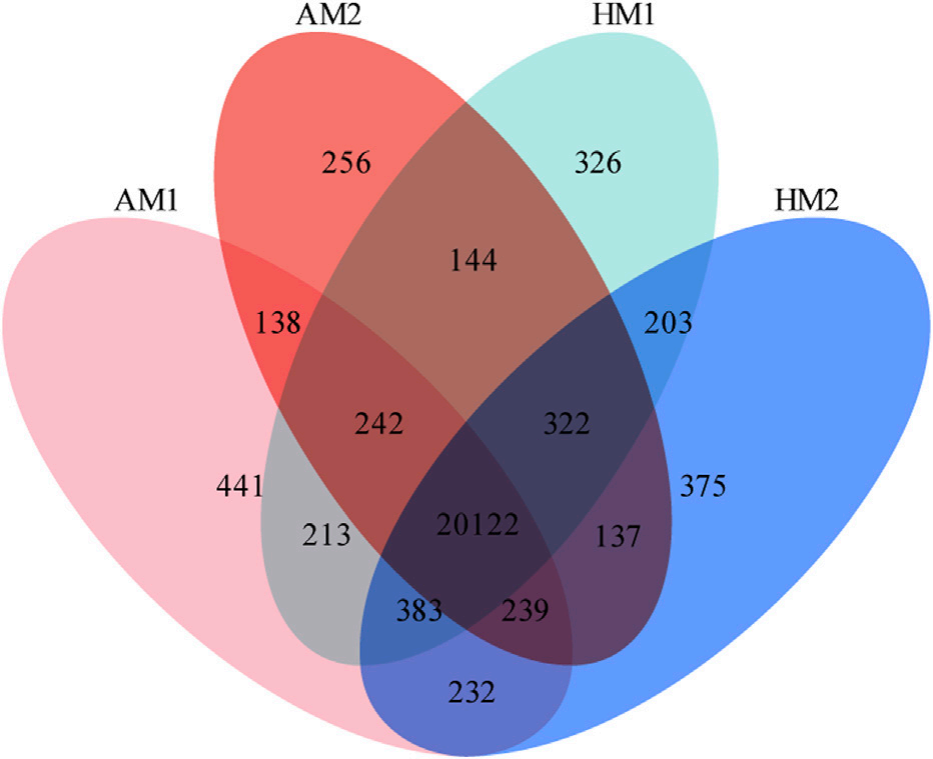
Venn diagrams of annotated genes in muscle tissue of Altay and Hu lambs exposed to different air temperatures. AM1: Altay lambs at −5°C; AM2: Altay lambs at 20°C; HM1: Hu lambs at −5°C; HM2: Hu lambs at 20°C.

A total of 23,773 genes with FPKM>0 were detected in all samples, including 20,624 known genes and 3,149 potentially novel genes (supplementary File 3). In total, 20,122 genes were co-expressed in all lambs, with 441, 256, 326, and 375 genes expressed only in AM1, AM2, AH1, and AH2 lambs, respectively (Figure 1).

### 3.3 Analysis of differential expression genes

**FIGURE 2:**
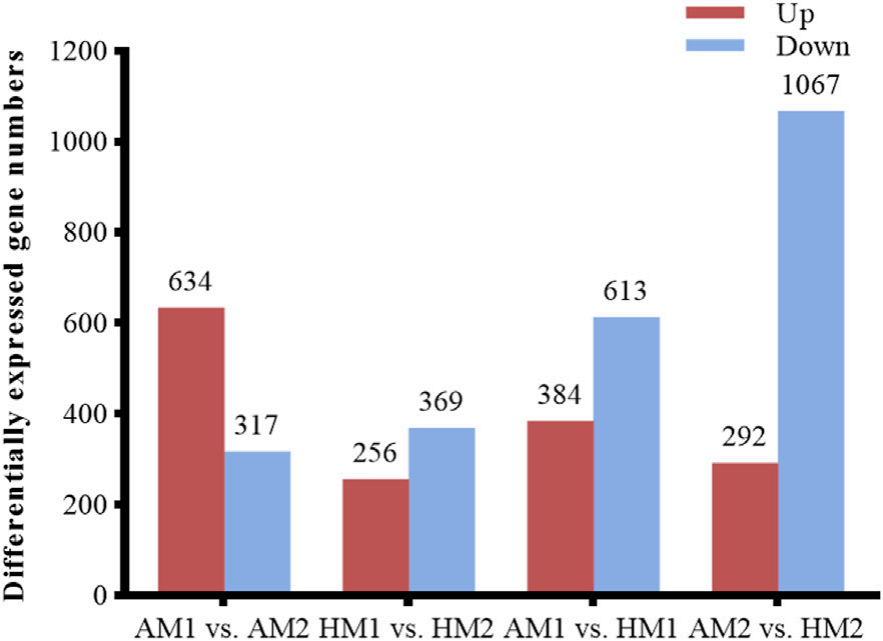
The number of up or down-regulated genes in Altay and Hu lambs. AM1 vs AM2: muscle tissue of Altay lambs at −5°C compared with lambs at 20°C; HM1 vs HM2: muscle tissue of Hu lambs −5°C compared with at 20°C; AM1 vs HM1: muscle tissue of Altay lambs at −5°C compared with Hu lambs at -5°C; AM2 vs HM2: muscle tissue of Altay lambs at 20°C compared with Hu lambs at 20°C.

**FIGURE 3:**
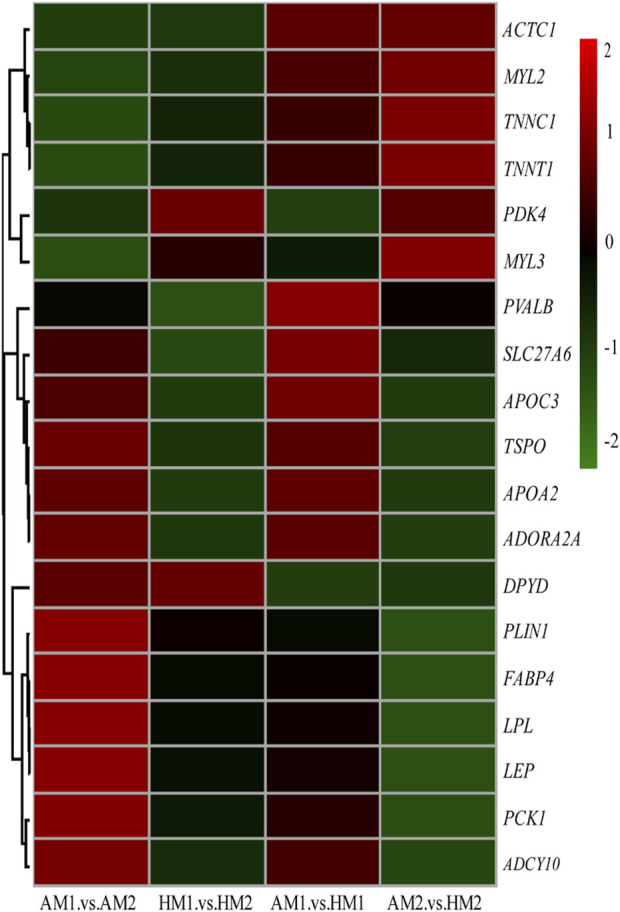
The heatmap of candidate genes in muscle tissue of Altay and Hu lambs. AM1 vs AM2: muscle tissue of Altay lambs at −5°C compared with lambs at 20°C; HM1 vs HM2: muscle tissue of Hu lambs −5°C compared with at 20°C; AM1 vs HM1: muscle tissue of Altay lambs at −5°C compared with Hu lambs at -5°C; AM2 vs HM2: muscle tissue of Altay lambs at 20°C compared with Hu lambs at 20°C.

After pairwise comparisons, the numbers of DEGs between different breeds or air temperatures are presented in Figure 2. The FPKM values of 634 DEGs were higher and of 317 genes were lower for Altay lambs at −5°C than at 20°C. The FPKM values of 256 genes of Hu lambs were up-regulated and 369 genes were down-regulated in lambs at −5°C than at 20°C. Compared with Hu lambs, in Altay lambs, 384 DEGs in -5°C lambs and 292 DEGs in 20°C lambs were up-regulated, whereas 613 genes in −5°C lambs and 1067 genes in 20°C lambs were down-regulated. The cluster heatmap of several candidate DEGs presented genes apparent expression difference in muscle tissue between lambs at −5°C and lambs at 20°C. The hereditary differences were substantial, as revealed by AM1 *versus* HM1 and AM2 *versus* HM2 (Figure 3).

**FIGURE 4:**
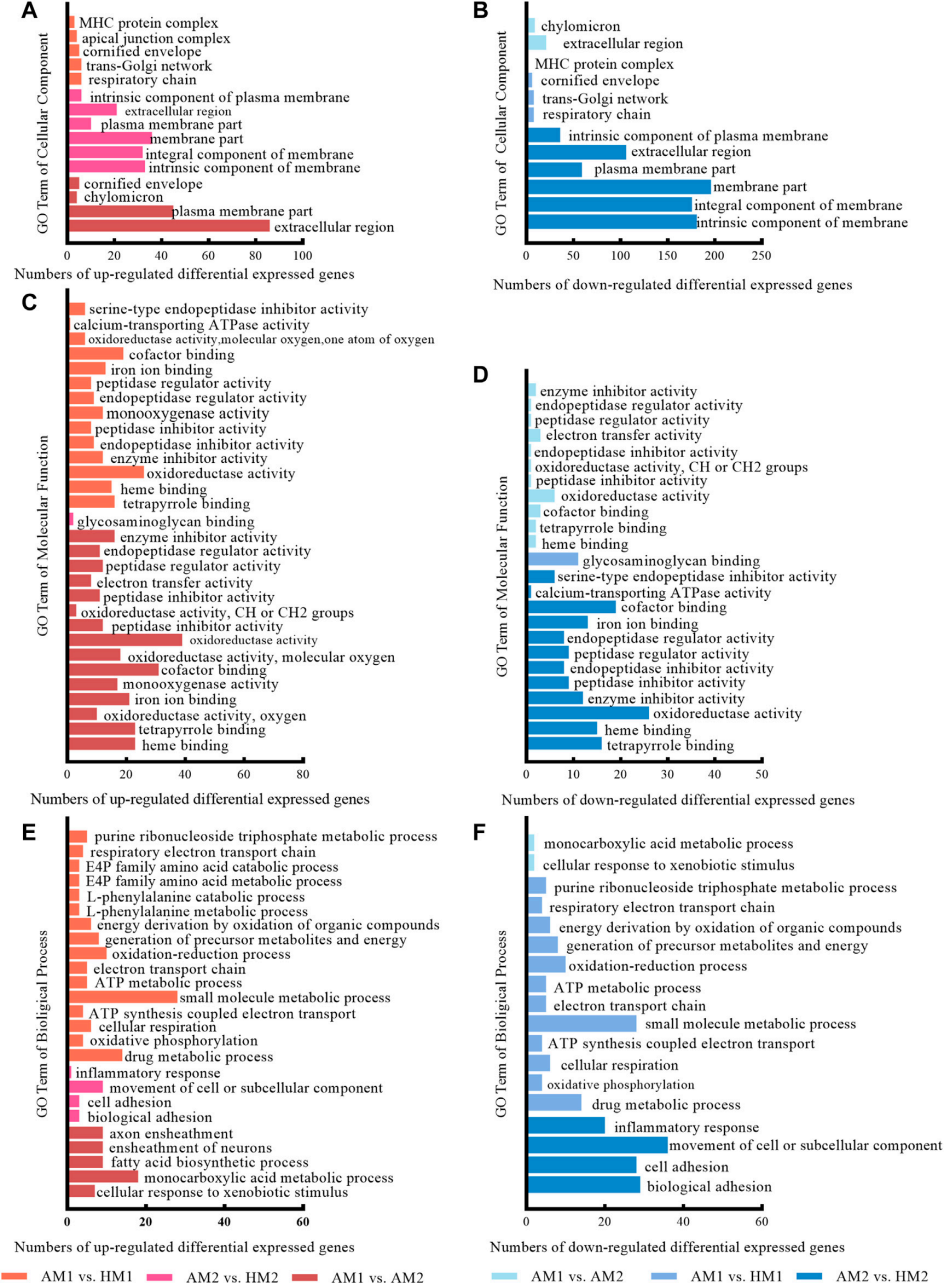
The significantly enriched gene ontology (GO) terms between Altay and Hu lambs at −5°C and 20°C in Altay and Hu breeds. AM1 vs. HM1: muscle tissue of Altay lambs at −5°C compared with Hu lambs at −5°C; AM2 vs. HM2: muscle tissue of Altay lambs at 20°C compared with Hu lambs at 20°C; AM1 vs. AM2: muscle tissue of Altay lambs at −5°C compared with lambs at 20°C.

The results of GO analysis illustrated that significant-change terms (corrected *p* < 0.05) were classified into cellular components, molecular functions, and biological processes (Figure 4).

The significant up-regulated and down-regulated terms of cellular components are presented in Figure 4A and Figure 4B, respectively. Most genes are in the extracellular region, plasma membrane part, and component of membrane, while few genes are in the chylomicron, respiratory chain, and MHC protein complex.

For molecular functions, significantly enriched terms included oxidoreductase activity, calcium-transporting ATPase activity, and co-factor binding (Figures 4C,D). For biological processes, terms including fatty acid biosynthetic process, oxidative phosphorylation, cellular respiration, ATP synthesis coupled electron transport, electron transport chain, and ATP metabolic processes, likely involved energy metabolism (Figures 4E,F).

To further identify the biological pathway, the DEGs were mapped in the KEGG pathway database, and all significantly changed (q-value≤0.05) pathways are presented in supplementary file 4. The PPAR and calcium signaling pathways changed significantly in Altay lambs at -5°C, while the calcium and cyclic AMP (cAMP) signaling pathways were enriched in Hu lambs at −5°C (Table 3). The DEGs in the calcium signaling pathway differed between Altay and Hu lambs.

**TABLE 3:** The annotated DEGs related to energy homeostasis in response to cold exposure in lambs.

KEGG pathway	Gene name	Gene id	Gene annotation	AM1 vs AM2	HM1 vs HM2
PPAR signaling pathway	*PCK1*	100037690	*phosphoenolpyruvate carboxykinase1*	Up-regulated	No difference
	*PLIN1*	100135431	*perilipin 1*	Up-regulated	No difference
	*FABP4*	100137067	*fatty acid binding protein 4, adipocyte*	Up-regulated	No difference
	*APOA2*	101104619	*apolipoprotein A2*	Up-regulated	No difference
	*SLC27A6*	101104743	*solute carrier family 27 members 6*	Up-regulated	No difference
	*APOC3*	101117273	*apolipoprotein C3*	Up-regulated	No difference
	*LPL*	443408	*lipoprotein lipase*	Up-regulated	No difference
Calcium signaling pathway	*BCAS1*	101102166	*breast carcinoma amplified sequence 1*	Up-regulated	No difference
	*CALN1*	101111598	*calneuron 1*	Up-regulated	No difference
	*CABP1*	101111598	*calcium binding protein*	Up-regulated	No difference
cAMP	*Fos*	443218	Fos	No difference	Up-regulated
	*TNMD*	100125616	*Tenomodulin*	No difference	Up-regulated
	*ADCY10*	101111248	*Adenylate Cyclase*	No difference	Down-regulated
	*ADORA2A*	101112926	*Adenosine A2a Receptor*	No difference	Down-regulated

AM1 vs AM2: muscle tissue of Altay lambs at −5°C compared with 20°C.

HM1 vs HM2: muscle tissue of Hu lambs at −5°C compared with at 20°C.

The results of KEGG pathways displayed substantial genetics differences between Altay and Hu lamb (Supplementary File S4). The calcium, cAMP, Rap1, Ras signaling pathways, and cardiac muscle contraction were enriched significantly in Altay lambs when compared with Hu lambs. In these pathways, seven co-expressed annotated genes, which were possibly involved with thermoregulation (Table 4), were higher in Altay than Hu lambs.

**TABLE 4:** The up-regulated differentially expressed genes (DEGs) related to energy homeostasis in Altay compared to Hu lambs.

KEGG pathway	Gene name	Gene id	Gene annotation	AM1 vs HM1	AM2 vs HM2
Calcium signaling pathway	*BDNF*	101117275	*brain-derived neurotrophic factor*	Up-regulated	Up-regulated
	*PVALB*	101122682	*Parvalbumin*	Up-regulated	Up-regulated
Cardiac muscle contraction	*TNNC1*	101111132	*troponin C1, slow skeletal and cardiac type*	Up-regulated	Up-regulated
	*MYL2*	100196904	*myosin light chain 2*	Up-regulated	Up-regulated
	*ACTC1*	101111554	*actin alpha cardiac muscle 1*	Up-regulated	Up-regulated

AM1 vs HM1: muscle tissue of Altay lambs at −5°C compared with Hu lambs at −5°C.

AM2 vs HM2: muscle tissue of Altay lambs at 20°C compared with Hu lambs at 20°

### 3.4 Analysis of protein-protein interaction

**FIGURE 5:**
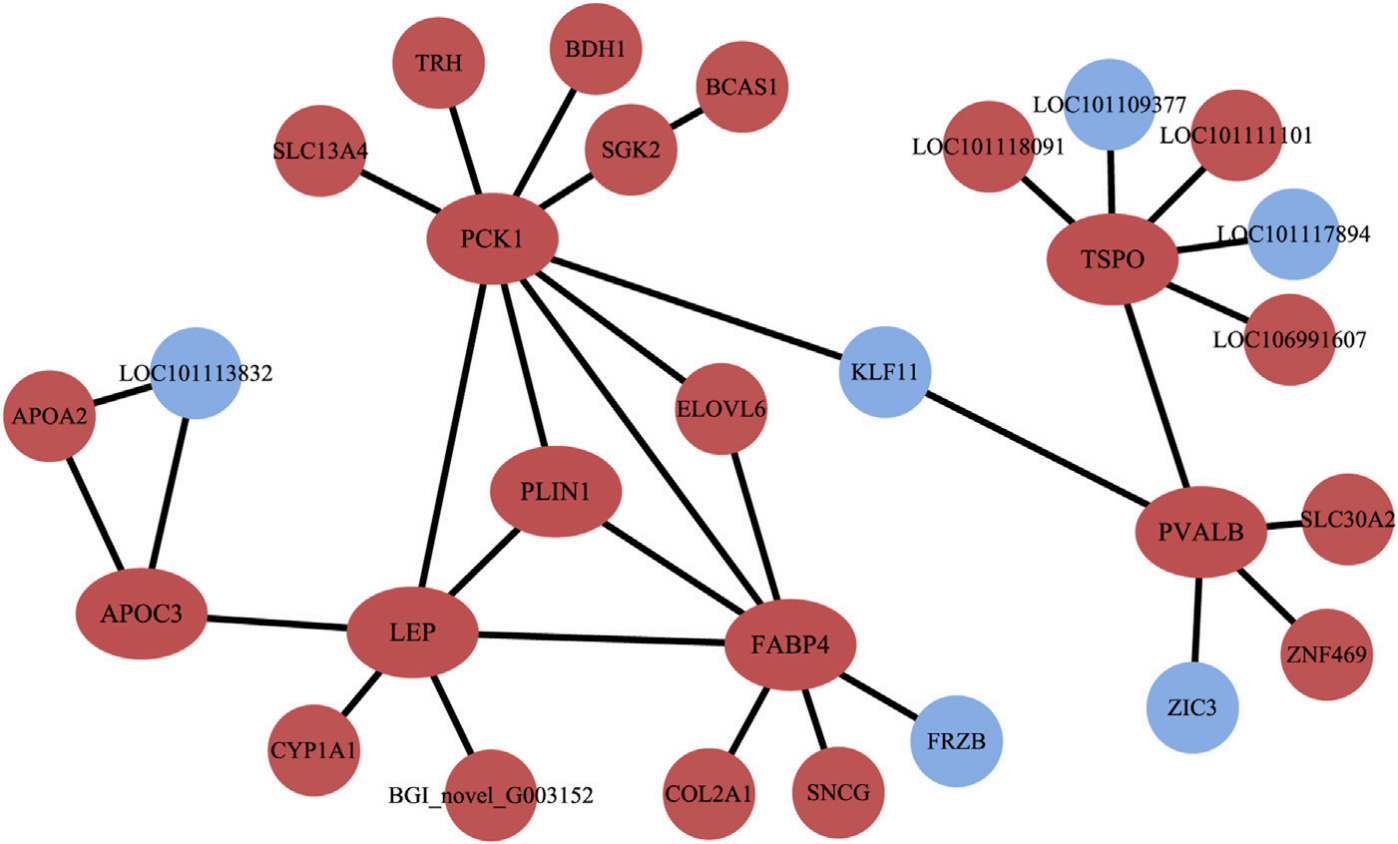
The interaction of protein coded by differentially expressed genes (DEGs). The red circles and ellipses represent up-regulated genes, while the wather blue circles represent down-regulated genes.

The PPI results illustrated a complex regulatory network (Figure 5). In Altay lambs at −5°C, the genes involved in the PPAR signaling pathway and muscle contraction increased, which contained *FABP4*, *LPL*, *PVALB*, *PCK1* (*phosphoenolpyruvate carboxykinase1*), *PLIN1* (*perilipin 1*), *APOA2* (*apolipoprotein A2*), *APOC3* (*apolipoprotein C3*), and *SLC27A6* (*solute carrier family 27 members 6*). Direct relations among their coding proteins, predicted by String Database, were detected (Figure 5). There was no regular PPI based on DEGs for cold-induced Hu lambs.

### 3.5 Validation of RNA-seq results by qRT-PCR

**FIGURE 6:**
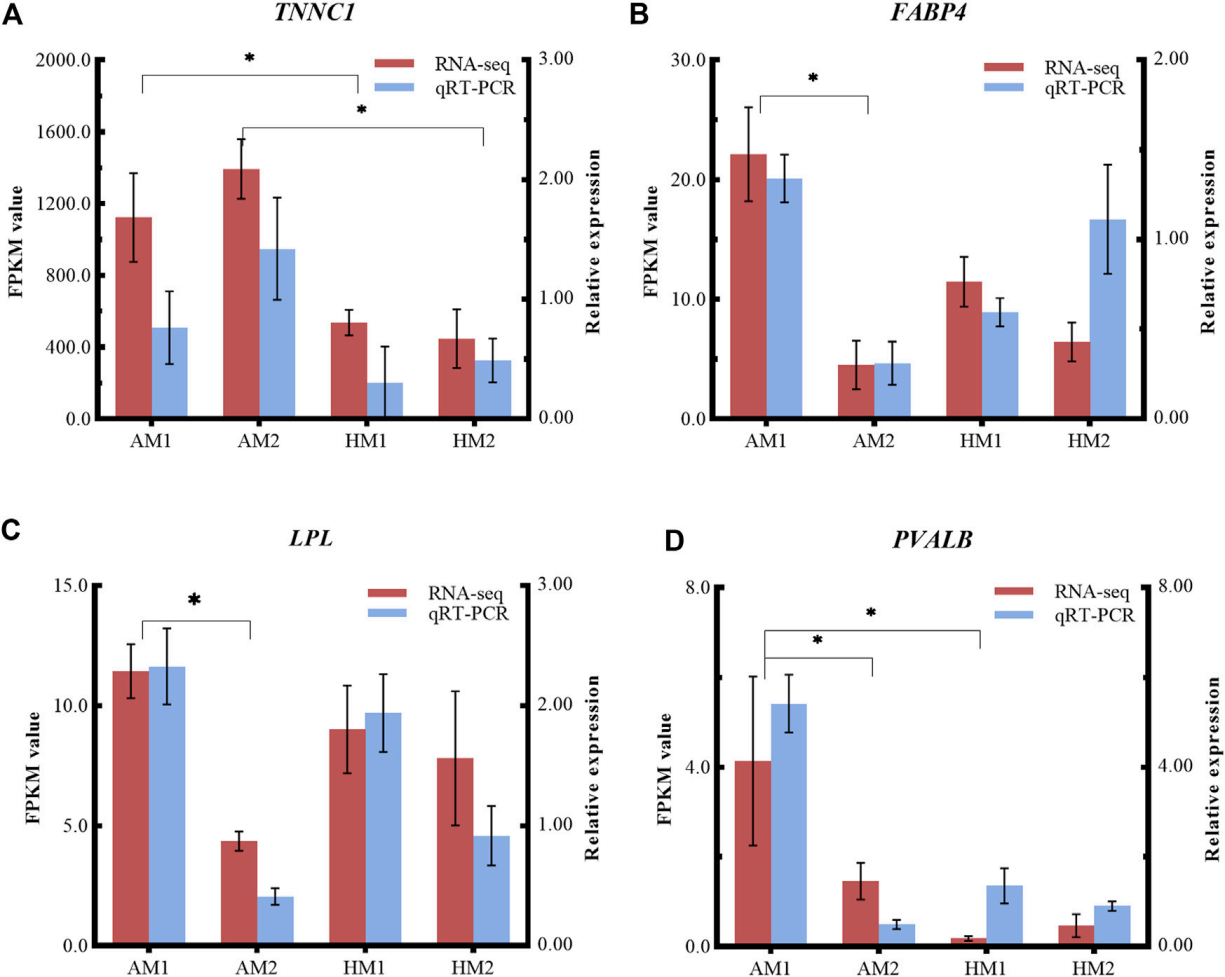
Relative expressions and fragments per kilobase of exon model per million reads mapped (FPKM) values from qRT-PCR and RNA-seq. The y-axis on the left and red bars represent the FPKM values of four genes from RNA sequencing. The y-axis on the right and blue bars represent the relative expressions of four genes from RNA-seq. The x-axis represents four groups in this study: **(A)** TNNC1; **(B)** FABP4; **(C)** LPL; and **(D)** PVALB. AM1: muscle tissue of Altay lambs at −5°C; AM2: muscle tissue of Altay lambs at 20°C; HM1: muscle tissue of Hu lambs at −5°C; HM2: muscle tissue of Hu lambs at 20°C.

To validate the RNA-seq results, four DEGs (*LPL, TNNC1*, *FABP4*, *PVALB*) were selected for qRT-PCR analysis (Figure 6). The consistent expression patterns between RNA-seq and qRT-PCR indicated that the expression profiles by RNA-seq were reliable.

## 4 Discussion

RNA sequencing with next-generation sequencing technology has provided a powerful, highly reproducible, and cost-effective tool (Xu et al., 2018). In recent years, transcriptome studies have reported specific molecular mechanisms and candidate genes for cold-exposed humans (Kim et al., 2021), mice (Shore et al., 2013), fish (Xu et al., 2018; Sun et al., 2019; Liu et al., 2020), gray treefrog (Amaral et al., 2020), and shrimp (Zhuo et al., 2020)*.* In the current study, we focused on skeletal muscle, as both ST and NST could contribute to systemic thermoregulation (Fuller-Jackson and Henry, 2018; Roesler and Kazak, 2020; Grigg et al., 2022).

Thermogenesis in muscle, and more so for NST, is regulated primarily at the protein expression level and by post-translational modifications, which was a limitation of the current study. Many studies have reported that NST from Ca^2+^ transport in muscle cells rely on RYR1 and SERCA, SLN and voltage-dependent anion channel (VDAC) (Maurya et al., 2018; Meizoso-Huesca et al., 2022), and the cold -induced protein expression of SERCA1 (Arruda et al., 2008; Sepa-Kishi et al., 2017) and SLN (Bal et al., 2017) increased. The current results showed the increased rectal temperature in cold-exposed Altay lambs and Hu lambs (Zhou et al., 2020) was likely due to activated UCP1-dependent thermogenesis involving the liver (Jiao et al., 2021a) and fat tissues (Jiao et al., 2021b), and UCP1-independent thermogenesis (calcium, PPAR and, cAMP signaling pathways) involving muscle tissue (present study). In further studies, we plan to carry out integrated analysis of proteomics, epigenetics and verification tests.

### 4.1 Genes involved in thermogenesis for cold-exposed altay sheep

#### 4.1.1 Calcium signaling pathway and DEGs

The calcium signaling pathway changed for both the Altay and Hu lambs. The gene expression of *BCAS1* (*breast carcinoma amplified sequence 1*), *CALN1* (*calneuron 1*) and *CABP1* (*calcium binding protein*) increased in cold-exposed Altay lambs. The calcium ion is a second messenger in cell excitation and excitation-contraction coupling in the myocyte, and the calcium signaling pathway is likely involved in producing heat. In cell contraction, passive calcium ions in the SR are transported into the cytoplasmic matrix by the ryanodine receptor (RYR). To maintain intracellular calcium homeostasis, calcium is propelled back into the SR *via* the SERCA against the concentration gradient (Fuller-Jackson and Henry, 2018). The hydrolysis of ATP mediated by SERCA is essential to this process, which, ultimately, produces heat (about 10–25 kcal/mol ATP) (Nowack et al., 2017). And thermogenesis is increased by SLN in muscle, as the N-terminus of SLN binding to SERCA could promote uncoupling of SERCA-mediated ATP hydrolysis from Ca^2+^ transport (Grigg et al., 2022). However, neither cold-exposed Altay or Hu lambs changed the expression of these key genes, that is, *SERCA1* (or *ATP2A1*), *RYR1*, *SLN*, and *VDAC1*. One possible reason is that they may contribute to thermogenesis (including NST) *via* coding protein and post-translational modifications, as reported difference in cold-induced protein expression in mice (Sepa-Kishi et al., 2017). Also, this may have occurred as a result of the specific muscle fiber type. The *longissimus dorsi* contains mainly fast-twitch type Ⅱ fibers that are highly glycolytic (Hamelin et al., 2007), like the *extensor digitorum longus* (EDL). In contrast, the soleus muscle is composed of more slow-twitch type I fibers that are highly oxidative. The muscle with slow- and fast-twitch fibers in cold-induced thermogenesis differ in their contractile apparatus, metabolic features, and protein level of SERCA (Wang et al., 2003; Wondmikun et al., 2003; Sepa-Kishi et al., 2017; Bal et al., 2021). From current reports, the up-regulated *CALN1* and *CABP1* code Ca^2+^-binding protein and have no thermogenic roles (Wu et al., 2001; Mccue et al., 2010; Kobuke et al., 2018; Mundhenk et al., 2019). Further studies are warranted on these DEGs in ruminants to understand their roles in thermoregulation.

#### 4.1.2 Peroxisome proliferator-activated receptors signaling pathway and DEGs

The PPAR signaling pathway in the muscle tissue of Altay lambs was altered in the present study. There are three PPARs, and all regulate glucose and fatty acid metabolism and energy homeostasis (Nakamura et al., 2014). PPAR-α, found mainly in the liver, is linked to β-oxidation of long chain fatty acids and ketogenesis (Takada et al., 2000; Gonzalez and Shah, 2008); PPAR-β/δ is highly expressed in skeletal muscle and is linked to oxidation of long chain fatty acids and glucose metabolism, and to biosynthesis of mitochondria; and PPAR-γ participates in the formation of triglycerides in adipose cells (Lehrke and Lazar, 2005; Nakamura et al., 2014). Glucose and free fatty acids are important substrates in generating heat in cold-exposed rodents (Paul and Holmes, 1973; Smith and Davidson, 1982; Sepa-Kishi et al., 2017). Non-esterified fatty acids, from the hydrolysis of triglycerides mediated by LPL, can be transported into cells by FABP4 to generate fat or produce heat *via* the tricarboxylic acid cycle (TCA) (Labbe et al., 2015). Extracellular glucose can also be utilized to generate heat after diffusing into the cell facilitated by glucose transporter 1–4 (Glut4) (Labbe et al., 2015; Garcia and Shaw, 2017)

In the present study, the expression of most DEGs, namely *PCK1*, *PLIN1*, *FABP4*, *APOA2*, *SLC27A6*, *APOC3*, and *LPL*, in the PPAR pathway increased in cold-exposed Altay. Some of these DEGs are likely involved in the metabolism of glucose and fatty acids in cells. PCK1 is a cytosolic isoenzyme of phosphoenolpyruvate carboxylase, and also an important rate-limiting enzyme that catalyzes the synthesis of phosphoenolpyruvic acid from oxaloacetate (Beale et al., 2007). Up-regulated PCK1 in muscle increased triglyceride concentration and running endurance, which implied that it could potentially contribute to efficiency and thermogenesis (Hakimi et al., 2007; Hanson and Hakimi, 2008). Both APOA2 and APOC3 are apolipoproteins, which are essential components of lipoprotein. They transfer high-density lipoprotein (HDL) in blood into specific tissues. PLIN1, a protein on the surface of lipid droplets, converts triglycerides into glycerol and free fatty acids through phosphorylation (Wen et al., 2011; Maurizi et al., 2017). In mice, *PLIN1* is a downstream target gene of PPAR-γ (Ying et al., 2015; Maurizi et al., 2017), and its depletion can cause abnormal expression of some genes related to fat metabolism (*CAAT/enhancer-binding proteins, sterol regulatory element-binding protein-1, FABP4*) and impair the ability to break down fat (Naoto et al., 2004). FABP4 is an important long-chain fatty acid binding transporter (Coe and Bernlohr, 1998; Labbe et al., 2015). In cold-exposed mice, the free fatty acids taken up by FABP4 are further esterified and transferred into the TCA cycle (Labbe et al., 2015), thereby, increasing heat production. In ruminants, FABP4 has been recognized as an important marker of intramuscular fat formation and its polymorphism was correlated with growth and carcass traits in livestock (Yan et al., 2018). SLC27A6, similar to FABP4, was linked to the transport of extracellular long-chain fatty acids, which can be used for heat production and fat synthesis (Stahl, 2004; Anderson and Stahl, 2013). Therefore, for thermoregulation at low air temperatures, it is likely that Altay sheep use glucose and fatty acid metabolism *via* the mediating gene differential expression in the PPAR signaling pathway.

In the current study, the rectal temperature of cold-exposed Altay lambs increased, however, the key genes for thermogenesis, such as *UCP3*, *SERCA1*, and *SLN*, did not. The elevated ATP production and Ca^2+^ concentration, induced by up-regulated genes in PPAR and the calcium signaling pathway, contributed the thermogenesis in muscle, despite the limited gene expression of *UCP*3, *SERCA1*, and *SLN*. In muscle during cold exposure, myosin ATPase and SERCA are the main heat producers that require ATP from mitochondria. Up-regulated DEGs in the PPAR signaling pathway could contribute to lipid metabolism, mitochondrial biogenesis and ATP production (Lim et al., 2015), which is also enhanced by calcineurin/cAMP response element-binding protein (CERB) or calcineurin/nuclear factor of activated T cells (NFAT) signaling cascade (Angus et al., 2005; Wu et al., 2006; Rotter et al., 2018). Both CALN1 and CABP1 serve as the calcium-binding receptor, and could increase Ca^2+^ level near SERCA and induce its ATP ultilization (Kobuke et al., 2018).

### 4.2 Genes involved in thermogenesis in cold-exposed hu sheep

The cyclic adenosine 3′,5′-monophosphate (cAMP) signaling pathway changed in cold-exposed Hu lambs. It was reported that intracellular cAMP deviated from the hydrolysis of ATP, mediated by adenylyl cyclase (AC) and some ions (for example, calcium or bicarbonate), with concomitant heat generation. Four annotated DEGs in the cAMP signaling pathway were identified. *Tenomodulin* (*TNMD*) and *Fos* were greater whereas *Adenylate Cyclase* (*ADCY10*) and *Adenosine A2a Receptor* (*ADORA2a*), which were linked to energy homeostasis, were lesser in cold-exposed lambs than the lambs at 20°C. *ADCY10* codes soluble adenylyl cyclase (sAC), which catalyzes the synthesis of cAMP by ATP from TCA and oxidative phosphorylation in intracellular mitochondria (Pozdniakova and Ladilov, 2018). ADORA2a is a member of adenosine receptor (AR) (Li et al., 2006; Rodrigues et al., 2014), and, in combination with BDNF, regulates heat production and energy balance in mice (Rodrigues et al., 2014). These results revealed that cAMP biosynthesis, mediated by the cAMP signaling pathway, plays a role in thermoregulation in cold-exposed Hu lambs.

### 4.3 Up-regulated genes in cold-exposed altay than hu lambs

Regardless of air temperature, calcium signaling pathways and cardiac muscle contraction differed between Altay and Hu lambs. Expressions of five potential genes, including *BDNF* (*brain-derived neurotrophic factor*), *PVALB*, *TNNC1*, *MYL2* (*myosin light chain 2*), and *ACTC1* (*actin alpha cardiac muscle 1*), were higher in muscle tissue of Altay lambs than Hu lambs.

The muscle contraction is mediated by troponin, myosin, actin, calcium ion and ATP (Li et al., 2004; Nowack et al., 2017). Ca2^+^ initially binds to troponin subunit C, which leads to the conformation change of troponin, and the exposure of the myosin cross-bridge binding site on the actin chain. Then actin, bound to the cross-bridge in myosin, activates ATP hydrolysis and contributes to heat generation (Li et al., 2004). Five proteins coded by the aforementioned genes most likely have important roles in this process. PVALB, a calcium ion buffering protein, regulates muscle relaxation by binding to Ca2^+^ (Zhang et al., 2017; Piorkowska et al., 2018), while TNNC1, MYL2 and ACTC1 are essential components of troponin, myosin, and actin, respectively (Parmacek and Solaro, 2005; Ploski et al., 2016; Squire et al., 2017). Importantly, MYL2 possesses intrinsic ATPase activity (Squire et al., 2017). BDNF is a neurotrophic protein factor that participates in maintaining mitochondrial function and metabolic homeostasis in muscle (Ahuja et al., 2022). The higher expression of these five annotated genes and higher rectal temperature (Zhou et al., 2020) in Altay lambs than Hulambs, suggests that muscle contraction contributed to maintaining body temperature.

## 5 Conclusion

**FIGURE 7:**
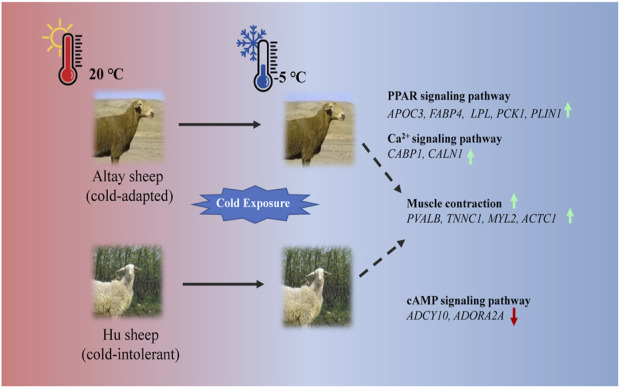
An illustration of genes involved in thermogenesis in cold-exposed Altay and Hu lambs. **(A)**: In cold-exposed Altay lambs, PPAR and calcium ion signaling pathway in muscle tissue were activated and differentially expressed genes (DEGs), such as APOC3, FABP4, LPL, PCK1, PLIN1, CABP, and CALN1 were up-regulated. **(B)**: In cold-exposed Hu lambs, cAMP signaling pathway was activated in muscle tissue and ADCY10, and ADORA2A were down-regulated. And the gene expression of PVALB, TNNC1, MYL2, and ACTC1 related to muscle contraction were higher in the muscle tissue of Altay than in Hu lambs.

In the present study, UCP1 independent pathways most likely participated in thermogenesis in cold-exposed sheep. In cold-exposed Altay lambs, PPAR signaling and calcium ion pathways in muscle tissue were activated and the genes, such as *APOC3*, *FABP4*, *LPL*, *PCK1*, *PLIN1*, *CABP1*, and *CALN1* were up-regulated. In cold-exposed Hu lambs; the cAMP signaling pathway was activated and *ADCY10* and *ADORA2A* were down-regulated. The gene expressions of *PVALB*, *TNNC1*, *MYL2*, and *ACTC1* related to muscle contraction were higher in muscle tissue of Altay than Hu lambs (Figure 7). These results shed light on the identification of genes in skeletal muscle that are associated with cold-tolerance in sheep.

## Data Availability

The datasets presented in this study can be found in online repositories. The names of the repository/repositories and accession number(s) can be found below: https://www.ncbi.nlm.nih.gov/, PRJNA858259.
