# Supplementary material for: Transcriptome analysis revealed potential genes involved in thermogenesis in muscle tissue in cold-exposed lambs
Source: Front Genet. 2022 Oct 21;13:1017458. doi: 10.3389/fgene.2022.1017458 (PMC9634817; doi:10.3389/fgene.2022.1017458)
Supplement: Supplementary file 3 [file Table1.DOCX]

Table S1. Chemical composition and energy content of alfalfa pellets

| Items | Concentration |
| --- | --- |
| Dry matter, g/kg | 902 |
| Organic matter, g/kg DM | 872 |
| Crude protein, g/kg DM | 148 |
| Neutral detergent fiber, g/kg DM | 464 |
| Acid detergent fiber, g/kg DM | 327 |
| Ether extract, g/kg DM | 16.8 |
| Gross energy, MJ/kg DM | 18.33 |

Table S2. Effect of chronic cold exposure on the rectal temperature in Altay sheep and Hu sheep

|  | -5 °C | |  | 20 °C | |  | *P*-value | | |
| --- | --- | --- | --- | --- | --- | --- | --- | --- | --- |
|  | Altay | Hu |  | Altay | Hu | SEM | Breed | Temperature | interaction |
| Rectal temperature, °C | | | | | | | | | |
| 06:00 h | 39.30 | 39.18 |  | 38.40 | 39.02 | 0.111 | 0.137 | <0.001 | 0.086 |
| 14:00 h | 39.17 | 39.23 |  | 38.92 | 38.77 | 0.076 | 0.093 | <0.01 | 0.120 |
| 22:00 h | 39.23 | 39.15 |  | 39.00 | 38.65 | 0.143 | 0.161 | 0.028 | 0.374 |
